# Congenital Central Hypoventilation Syndrome: A Case Report

**DOI:** 10.7759/cureus.64884

**Published:** 2024-07-19

**Authors:** Gaurav Kumar, Shiji Chalipat, Sudhir Malwade, Sanjay Chavan, Sanika Pimparkar

**Affiliations:** 1 Pediatrics, Dr. D. Y. Patil Medical College, Hospital and Research Centre, Dr. D. Y. Patil Vidyapeeth (Deemed to be University), Pune, IND; 2 Pediatric Neurology, Dr. D. Y. Patil Medical College, Hospital and Research Centre, Dr. D. Y. Patil Vidyapeeth (Deemed to be University), Pune, IND; 3 Pediatrics and Neonatology, Dr. D. Y. Patil Medical College, Hospital and Research Centre, Dr. D. Y. Patil Vidyapeeth (Deemed to be University), Pune, IND

**Keywords:** polyalanine repeats, central congenital hypoventilation, apnea, congenital central hypoventilation syndrome, central hypoventilation

## Abstract

Congenital central hypoventilation syndrome (CCHS) is a rare cause of apnea and hypoventilation requiring long-term multidisciplinary care. In this article, we report the case of a two-month-old female child who presented with recurrent apnea and cyanosis, requiring long-term ventilation. After ruling out other common causes of apnea like sepsis, metabolic disorders, and neuromuscular disorders, a genetic study was done, which confirmed the diagnosis of CCHS. The child was discharged on home oxygen therapy, and the parents were counseled about genetic testing and informed about the prognosis and requirement for home ventilation therapy, as well as parental testing.

## Introduction

Congenital central hypoventilation syndrome (CCHS) is an extremely rare disorder characterized by abnormal regulation of breathing by the autonomic system, leading to alveolar hypoventilation, particularly during deep, slow-wave sleep. The incidence is 1/148000-1/200000 live births, and it presents in infancy with repeated episodes of apnea or cyanosis, sometimes cardiorespiratory arrest [1]. These patients lack the response to hypercapnia and hypoxemia and, therefore, do not adequately augment respiratory effort during ventilatory challenges [2].

Due to autonomic dysfunction central to CCHS, there are deficiencies in the central integration of chemoreceptor inputs, which are responsible for the loss of respiratory drive in CCHS [3]. This disrupted response to hyperoxia causes altered responses in the amygdala, which normally regulates respiratory function [4].

CCHS is an uncommon lifelong condition, previously referred to as Ondine's curse, which was first described by Mellins et al. in 1970 [5]. The disease-defining gene of CCHS was discovered in 2003, and it was the paired homeobox 2B (PHOX2B) gene on chromosome 4p12, which is vital for the migration of neural crest cells and the development of the autonomic nervous system (ANS) [6,7]. The age of presentation can be neonatal or late-onset (>1 month of age, childhood, or adulthood) with no definite sex predilection. Usually, these infants have perinatal asphyxia, requiring neonatal resuscitation [8]. Most children with CCHS require early tracheostomy and home ventilation to prevent significant hypoxia and increase survival. The frequency of apneic episodes in children with CCHS decreases with age, and some patients may survive into adulthood [9].

We describe a case of an infant with genetically proven CCHS with recurrent episodes of apnea requiring long-term assisted ventilation.

## Case presentation

A two-month-old girl presented to the emergency department with recurrent episodes of apnea and cyanosis. She was born at term via normal vaginal delivery, weighing 3 kg, and had an uneventful perinatal period. However, on day 18 of life, she was admitted to the Neonatal Intensive Care Unit for respiratory distress and required invasive ventilation. Despite a thorough evaluation, the cause of her respiratory distress remained unclear. The child experienced multiple admissions for recurring apneas, necessitating mechanical ventilation almost continuously at an outside hospital. At two months old, she was referred to our institute, where she was intubated and mechanically ventilated.

On examination, the patient's head circumference was 38 cm (between the median and -1 standard deviation). She exhibited subtle dysmorphic features, including a long philtrum, a square face, retrognathia, and hypertelorism. The baby had global hypotonia but well-elicitable deep tendon reflexes. The remainder of the systemic examination was normal.

Blood investigations at the time of admission were all within the normal limits except for a for a low level of hemoglobin, a low hematocrit, and slightly elevated creatinine (Table 1).

**Table 1:** Blood investigations CPK NAC: creatine phosphokinase N-acetyl cysteine

Parameter	Observed value	Normal range
Hemoglobin	8.5 gm/dl	11-14gm/dl
Total leucocyte count	9100/uL	5000-12000/ uL
Differential leucocyte count (N/L)	60/28 %	-
Platelet count	2,01,000/uL	1,50,000-4,10,000/uL
Hematocrit	25.4 %	33-42 %
Urea	44 mg/dl	17-49 mg/dl
Creatinine	0.55 mg/dl	0.17-0.49 mg/dl
Sodium	146 mmol/L	139-146 mmol/L
Potassium	3.9 mmol/L	3.5-5.10 mmol/L
Chloride	104 mmol/L	98-107 mmol/L
Calcium	9.1 mg/dl	9-11 mg/dl
Ionic calcium	1.11 mmol/L	1.1-1.32 mmol/L
Magnesium	2 mg/dl	1.8-2.4 mg/dl
CPK NAC	42 U/L	26-192U/L
Blood sugar	98 mg/dl	-

Arterial blood gas analysis revealed respiratory acidosis with compensated metabolic alkalosis (Table 2). A bedside electroencephalogram was conducted to rule out seizures and was found to be normal. The nerve conduction test showed normal conduction velocity and amplitude, and an electromyography study was also normal. The negative neostigmine test and normal repetitive nerve stimulation test ruled out congenital myasthenia gravis. Cardiac evaluation and neuroimaging were both normal. Metabolic screening showed normal ammonia and lactate levels, and gas chromatography, mass spectrometry, and tandem mass spectrometry results were normal.

**Table 2:** ABG analysis HCO3: bicarbonate, PaO2: partial pressure of oxygen, PaCO2: partial pressure of carbon dioxide, ABG: arterial blood gas

Parameter	Value	Normal range
pH	7.25	7.35-7.45
HCO3-	32 mEq/L	22-28 mEq/L
pCo2	50.4 mmHg	35-45 mmHg
pO2	274 mmHg	75-100 mmHg

Given the normal results of all investigations, differential diagnoses included CCHS and congenital neuromuscular disorders. Clinical exome sequencing was arranged to investigate these possibilities further. Clinical exome sequencing identified a heterozygous mutation in the PHOX2B gene on chromosome 4p12 (transcript NM_0003924 3, variant c.753_767dupGGCGGCGGCAGCGGC(P.Ala256_Ala260dup) in exon 3), indicating an autosomal dominant inheritance pattern. The parents were counseled about the genetic findings, the prognosis, and the necessity for home ventilation therapy. Parental genetic testing was also advised.

In the pediatric intensive care unit, the baby received ventilatory support and oxygen therapy. Despite repeated extubation failures, the baby was successfully extubated on the third attempt on the 18th day. After extubation and stabilization, the child was gradually transitioned to nasogastric feeds and eventually sent home on oral feeds and home oxygen therapy.

## Discussion

CCHS is a unique lifelong disorder, previously described as Ondine's curse, which was first described by Mellins et al. in 1970 [5]. The disease-defining gene of CCHS was discovered in 2003, and it was the PHOX2B gene on chromosome 4p12. This gene is an important regulator of neuronal crest migration and ANS development [6,7]. The PHOX2B gene has two polyalanine repeat regions in exon 3. Studies have shown that polyalanine repeat mutations (PARM) are associated with decreased transcription of genes. More than 92% of patients with CCHS have PARMs in the PHOX2B gene, ranging from 24 to 33 alanine. The length of the expansion determines the severity of the disease. Our patient had a genetic mutation on the PHOX2B gene on PARMs of 21 repeats. This variant has been reported previously by Shimokaze et al. and has been associated with a less severe clinical phenotype [10].

Many of the infants will have perinatal asphyxia requiring neonatal resuscitation, whereas in our case, the perinatal course was uneventful [8]. There is no definite sex predilection seen, even though a study from Japan by Shimokaze et al. has reported a male predominance in infants with 25 PARM [10]. The age of presentation can be neonatal or late-onset (>1 month of age, childhood, or adulthood).

Most children with neonatal-onset CCHS present within the first 48 hours of life. Our patient presented on day 18 of life, which, while still within the neonatal period, is later than typical. It is possible that the parents did not notice subtle signs of duskiness during sleep in the initial days. Common presentations include episodes of cyanosis, apnea, or even cardiorespiratory arrest [1].

Todd et al. described distinct facial characteristics in children with CCHS, which include a square face with a tall, flat forehead, a deep philtrum, and downturned lips [11]. These features are frequently observed in children with PARMs in the PHOX2B gene. Our patient also exhibited several of these facial features consistent with PARM repeats. Infants with CCHS may also exhibit other signs of autonomic dysfunction, such as absent or sluggish pupillary reflexes, strabismus, and cardiac conduction abnormalities or arrhythmias. However, our patient did not show any of these additional manifestations [7].

Our patient experienced more episodes of apnea during sleep and occasional episodes while awake. Blood gas analysis revealed carbon dioxide retention with respiratory acidosis and compensated metabolic alkalosis, which are classical findings in CCHS. We ruled out sepsis and metabolic causes, and a detailed workup for neuromuscular etiologies was negative. Normal neuroimaging ruled out posterior fossa and brainstem malformations. Although approximately 20% of children with CCHS have Hirschsprung disease, our patient did not [7].

Most children with CCHS require early tracheostomy and home ventilation to prevent significant hypoxia and increase survival. Despite the recommendation for home ventilation, our patient's family faced financial constraints and left the hospital against medical advice, opting for home oxygen therapy instead. Previous studies indicate that home oxygen therapy is not effective in managing CCHS. With advancements in technology, mechanical ventilation through CPAP or BIPAP can be administered at home [9]. Reported literature suggests that the frequency of apneic episodes in children with CCHS decreases with age, and some patients may survive into adulthood. Respiratory stimulants have no proven role in the management of CCHS. Given the potential for parental mosaicism, parental genetic testing is advised to prevent recurrence in future offspring [9].

## Conclusions

This case highlights the clinical challenges and diagnostic journey of managing a two-month-old girl with CCHS who presents with recurrent apnea and cyanosis. Despite an initially unclear etiology, thorough genetic testing confirmed the presence of a PHOX2B gene mutation. The patient's clinical management underscores the importance of early recognition, genetic counseling, and the necessity for specialized home ventilation support. Advancements in genetic understanding and technology continue to improve the prognosis and quality of life for individuals with CCHS, emphasizing the need for continued research and support for affected families.
